# Molecular background of Leber congenital amaurosis in a Polish cohort of patients—novel variants discovered by NGS

**DOI:** 10.1007/s13353-022-00733-9

**Published:** 2022-11-12

**Authors:** Anna Skorczyk-Werner, Anna Sowińska-Seidler, Anna Wawrocka, Joanna Walczak-Sztulpa, Maciej Robert Krawczyński

**Affiliations:** 1 Department of Medical Genetics, Poznan University of Medical Sciences, Poznan, Poland; 2 Centers for Medical Genetics GENESIS, Poznan, Poland

**Keywords:** Leber congenital amaurosis (LCA), Inherited retinal dystrophies (IRDs), Whole exome sequencing (WES), Targeted next-generation sequencing (NGS), Novel variants

## Abstract

Leber congenital amaurosis (LCA) is the most severe form of inherited retinal dystrophies and the most frequent cause of congenital blindness in children. To date, 25 genes have been implicated in the pathogenesis of this rare disorder. Performing an accurate molecular diagnosis is crucial as gene therapy is becoming available. This study aimed to report the molecular basis of Leber congenital amaurosis, especially novel and rare variants in 27 Polish families with a clinical diagnosis of LCA fully confirmed by molecular analyses. Whole exome sequencing or targeted next-generation sequencing (NGS) of inherited retinal dystrophies-associated (IRD) genes was applied to identify potentially pathogenic variants. Bidirectional Sanger sequencing and quantitative PCR (qPCR) were carried out for validation and segregation analysis of the variants identified within the families. We identified 28 potentially pathogenic variants, including 11 novel, in 8 LCA genes: *CEP290*, *CRB1*, *GUCY2D*, *NMNAT1*, *RPGRIP1*, *CRX*, *LRAT1*, and *LCA5.* This study expands the mutational spectrum of the LCA genes. Moreover, these results, together with the conclusions from our previous studies, allow us to point to the most frequently mutated genes and variants in the Polish cohort of LCA patients.

**Supplementary Information:**

The online version contains supplementary material available at 10.1007/s13353-022-00733-9.

## Introduction

Leber congenital amaurosis (LCA) is a rare retinal disorder, classified in a group of inherited retinal dystrophies (IRD). LCA is the most severe form of IRD and the most frequent cause of congenital blindness in children. The disease accounts for about 5% of all IRDs. The prevalence of LCA is estimated to be 1 in 30,000 births (Koenekoop 2004). The disease typically manifests in the first year of life, but LCA is genetically and phenotypically heterogeneous. The symptoms usually include nystagmus, severe, and early visual impairment, Franceschetti’s oculo-digital sign (comprising eye-poking, pressing, and rubbing), and very often the absence of fixation in infants. Other symptoms of LCA are photophobia, refraction defects, night blindness, keratoconus, and cataract. A typical finding that defines LCA is severely reduced or extinguished full-field electroretinography (ERG) responses (Kumaran et al. 2018). The rate of visual loss ranges from functional visual acuity to light perception or even total blindness. Funduscopic imaging may show a normal fundus, especially in small children, and a variety of retinal pigment rearrangements involving peripheral retinopathy, vascular attenuation, and central maculopathy. Patients with a normal fundus appearance in the first 2 years usually develop pigmentary retinopathy, optic disc pallor, and vascular attenuation with time (Kumaran et al. 2018; Huang et al. 2021).

The course of the disease and symptoms are sometimes variable and often very similar to these of other IRDs. This makes it difficult to diagnose Leber congenital amaurosis, which is often confused, e.g., with retinitis pigmentosa. In many cases, only the results of genetic-molecular testing may allow for the diagnosis of LCA to be confirmed. To date, 25 genes are known to be implicated in the pathogenesis of LCA (https://sph.uth.edu/retnet/). Determination of the molecular basis of the disease allows for the identification of potential gene candidates for treatment.

The study aimed to report the molecular basis of Leber congenital amaurosis, especially novel and rare variants in 27 Polish families with a clinical diagnosis of LCA, fully confirmed by molecular analyses. Moreover, these results, together with the conclusions from our previous studies, allow us to point to the most frequently mutated genes and the most popular variants in the Polish cohort of LCA patients.

## Materials and methods

### Patients

Altogether, 66 families with a clinical diagnosis of LCA, who were referred to our genetic clinic from 2010 to 2021, were subjected to molecular testing. In our previous study (Skorczyk-Werner et al. 2020), we described a group of 22 families with a diagnosis of LCA confirmed by the results of the LCA SNP microarray and NGS-LCA panel. In this study, we present a group of another 27 Polish families with LCA molecular diagnosis established based on WES or targeted NGS of IRD-associated genes panel. The remaining 17 out of 66 families have not received a full molecular diagnosis and these patients are not described in this study.

### Clinical diagnosis of LCA

A total of 31 patients from 27 unrelated Polish families affected with LCA confirmed by molecular analysis results were evaluated in this study. All patients were referred to ophthalmologic examination including best-corrected visual acuity (BCVA) and funduscopy. Electroretinography (ERG) was performed on 24 patients. ERG was not available for patients: 26–80, 26–81, 28–89, 29–92, and 45–161 due to strong nystagmus. ERG was also not performed in patients: 38–146 and 37–144. Fundus autofluorescence was conducted in patients: 29–92, 42–158, 43–159, and 48–166. Optical coherence tomography (OCT) was performed in patients: 34–117, 34–118, 39–154, 42–158, 47–164, and 48–166. Magnetic resonance (MR) of the head and orbits was performed during early infancy in five patients: 40–156, 42–18, 45–161, 46–163, and 49–167. The symptoms observed in a group of 31 patients characterized in this study are listed in Table 1.

**Table 1:** Clinical symptoms and the results of the ophthalmological examinations in 27 Polish families with LCA

Patient ID^1^	Current age (years), gender	Disease onset and first symptoms	BCVA OD OS	Fundus appearance (age at the funduscopy)	Other ophthalmological signs and symptoms	ERG results	Other non-ocular symptoms
23–66	31, F	1 month—nystagmus, no fixation, no pacing, absent pupillary responses	Light perception	Blurred fundus image due to cataract, possibly pale optic nerve heads (17 years)	Cataract of both eyes, keratoconus of both eyes	Extinguished	-
24–72	33, F	After birth—oculodigital sign, 2 months—nystagmus, no fixation, no pacing, absent pupillary responses	No light perception from early childhood	Pale optic nerve heads (2 months)	-	Extinguished	-
25–76	28, F	3 months—nystagmus, photophobia	0.5/50 (9 years)	Impossible to perform due to strong nystagmus	High hyperopia	Extinguished	-
25–77	23, F	After birth—nystagmus, oculodigital sign, sluggish pupillary responses	Counting fingers	Thin retina, attenuated vessels, a dispersed pigment in the macula (15 years)	-	Extinguished	-
26–80	55, M	After birth—nystagmus, photophobia, congenital cataract, deterioration of color vision	No light perception	Thin retina, retinal pigment deposits, pale optic nerve heads (46 years)	-	Impossible to perform due to strong nystagmus	-
26–81	32, F	After birth—nystagmus, congenital cataract, deterioration of color vision	Light perception	Pale optic nerve heads, salt, and pepper fundus appearance (23 years)	Keratoconus of both eyes	Impossible to perform due to strong nystagmus	-
27–85	36, M	1 year—nystagmus, photophobia, night blindness (from the age of 3), deterioration of color vision, gradual visual field constriction	Hand movements	Oval retinal pigment deposits covering macula also (21 years)	-	Extinguished (3 years)	-
28–88	15, F	After birth—nystagmus, oculodigital sign, no fixation, no pacing, photophobia	No light perception	No fundus changes (2 months); dispersed retinal pigment deposits (2 years)	Deep-set eyes, high hyperopia	Extinguished (2 years)	Mild midface hypoplasia
28–89	9, M	3 months—nystagmus, no fixation, no pacing, light photophobia 6 months—the oculo-digital sign, sluggish pupillary responses,	No light perception	Normal fundus appearance (6 months)	Photophobia, high hyperopia	Impossible to perform due to strong nystagmus	-
29–92	42, F	3 months—nystagmus, photophobia, deterioration of color vision, irregular visual field loss	Hand movements	Retinal pigment deposits in the macula, peripheral bone-spicule pigmentation, pale optic nerve heads, attenuated vessels, focal RPE atrophy (28 years)	Keratoconus of both eyes	Impossible to perform due to strong nystagmus	midface hypoplasia
30–93	21, F	2 months—nystagmus, photophobia, oculodigital sign, no fixation, no pacing, no eye contact, sluggish pupillary responses	Light perception (intense light only)	Attenuated vessels, irregular pigment deposits, peripheral bone-spicule pigmentation, atrophic optic nerve heads (10 years)	High hyperopia, keratoconus	Extinguished	-
31–102	23, M	2 months—nystagmus, strabismus, no fixation, no pacing, no eye contact	Light perception (intense light only)	Attenuated vessels, optic nerve heads drusen (17 years)	Pseudophakia of both eyes, after cataract extraction (17 years)	Extinguished	-
32–107	4, M	3 months—nystagmus, oculodigital sign, no fixation, no pacing, no eye contact	Poor light perception	Retinal pigment deposits, attenuated vessels, loss of macular reflex (2 years)	High hyperopia with astigmatism	Extinguished	-
33–110	12, F	2 months—nystagmus, no fixation, no pacing, no eye contact, sluggish pupillary responses, photophobia, strabismus	Light perception (strong light only)	Dispersed retinal pigment deposits, attenuated vessels (8 years)	Deep-set eyes	Extinguished	-
34–118	29, F	2 months—nystagmus, oculodigital sign, sluggish pupillary responses, photophobia, deterioration of color vision, night blindness	Light perception	Retinal pigment rearrangements and yellowish deposits, salt and pepper fundus, attenuated vessels (1 year)	Deep-set eyes	Extinguished	-
34–117	21, F	3 months—nystagmus, night blindness	Light perception	Attenuated vessels, atrophic optic nerve heads, salt, and pepper fundus (19 years)	-	Extinguished	-
35–119	3, M	After birth: nystagmus, sluggish pupillary responses	No light perception	Pale optic nerve heads, hypoplastic macula, dispersed, fine-grained retinal pigment deposits (2 years)	-	Extinguished	Delayed psychomotor development, prominent forehead, high hairline, low set ears, high palate*
36–133	3, F	3 months—nystagmus, oculodigital sign, no fixation, no pacing, no eye contact	No light perception	Normal fundus appearance (4 months)	-	Extinguished	-
37–144	21, M	2 months—nystagmus, oculodigital sign, no fixation, no pacing, no eye contact	Hand movements	Pale optic nerve heads, dilatation of the disc vessels, few telangiectasias in the retina, dispersed, fine-grained retinal pigment deposits (21 years)	High hyperopia with astigmatism, bilateral keratoconus (after corneal transplantations) (LE −16 years, RE −19 years), cataract, secondary glaucoma	Not performed	-
38–146	6, M	5 months—nystagmus	1/50 2/50	Loss of macular reflex, retinal pigment deposits in the macula (6 years)	High hyperopia (+9.0 D)	Not performed	-
39–154	49, F	2 months—photophobia, nystagmus, peripheral visual field loss	Hand movements	Pale, atrophic retina with numerous, round retinal pigment deposits (“leopard skin” appearance)	High hyperopia	Extinguished	-
40–156	3, F	2 months—nystagmus, oculodigital sign, no fixation, no pacing, no eye contact, sluggish pupillary responses	Difficult to assess	Pale optic nerve heads, no macular reflex, small degenerative changes in and around the macula with hypo- and hyperpigmentation of the RPE, attenuated vessels (2 years)	High hyperopia (+8.0 D) with astigmatism	Extinguished	-
41–157	7, F	2 months—nystagmus, no fixation, no pacing, no eye contact	2/50	Pale optic nerve heads, beige retina, pigment deposits in macula, attenuated vessels (6 years)	High hyperopia with astigmatism, night blindness	Extinguished	-
42–158	11, M	1 year—decreased BCVA, strabismus, nystagmus	2/50	Normal fundus appearance on fundoscopy; bull’s eye maculopathy on fundus autofluorescence (FAF) (10 years)	Hyperopia (+3.0 D), nystagmus	Extinguished	Developmental disorder and motor hyperactivity
43–159	14, M	2 years—nystagmus, photophobia, reduced visual acuity, deterioration of color vision, night blindness	5/50	Peripheral bone-spicule pigmentation, pale optic nerve heads, attenuated vessels (14 years)	High hyperopia (+6.0 D)	Diminishes responses	-
			5/25				
44–160	7, F	2 months—nystagmus, strabismus, hyperopia	1/50	Pale optic nerve heads, attenuated vessels, thin retina, numerous retinal pigment deposits, bull’s eye maculopathy (5 years)	Hyperopic astigmatism (+8.0 D)	Extinguished	-
45–161	2, F	3 months—nystagmus, sluggish pupillary responses, no fixation, no pacing, no eye contact	Difficult to assess	Normal fundus appearance (3 months)	High hyperopia (+9.0 D)	Impossible to perform due to strong nystagmus	Slightly delayed psychomotor development
46–163	1, F	3 months—nystagmus, no fixation, no pacing, no eye contact, oculo-digital sign	Difficult to assess	Normal fundus appearance (3 months)	-	Scotopic—residual, photopic extinguished	-
47–164	34, F	After birth: photophobia, later: irregular visual field loss, color vision deterioration	2/50	Thin retina, peripheral and macular retinal pigments rearrangements with RPE atrophy, (45 years)	High hyperopia (+9.0 D) with astigmatism,	Photopic—residual, scotopic-significantly reduced	-
48–166	10, F	2 years: strabismus, hyperopia, later: nystagmus, night blindness, tunnel visual field	Almost normal visual acuity with a periodic decline	Dispersed retinal dystrophic changes in the central and peripheral retina (10 years)	Hyperopia, visual field constricted to 5^o^	Extinguished	-
49–167	1, M	2 months—nystagmus, no fixation, no pacing, no eye contact, oculodigital sign	Difficult to assess	Normal fundus appearance (6 months)	Wandering eye movements, sluggish pupillary responses	Extinguished	-

*BCVA* best corrected visual acuity, *RE* right eye, *LE* left eye, *M* male, *F* female, *–* not present

^1^The first digit indicates the family number and the next digit after the hyphen refers to the laboratory number of the individual

*Dysmorphic features typical to MidX28 syndrome, identified in this patient based on arrayCGH

### Molecular genetic analysis

DNA samples from the affected individuals, their healthy parents, and unaffected siblings (in families: 31, 36, 38, 41, and 42) were extracted for genetic examination. The total number of samples was 80. In most patients and their families, genomic DNA was extracted from the venous blood using the MagCore extractor system H16 with a MagCore Genomic DNA Whole Blood Kit (RBC Bioscience Corp., Taiwan). In three families (no. 40, 41, and 44), DNA samples from the proband’s relatives, for the segregation analysis, were extracted from buccal swabs according to a kit protocol (Nucleo-Spin Tissue, Machery-Nagel).

Whole exome sequencing or targeted next-generation sequencing (NGS) of IRD-associated genes was performed on DNA samples of one proband from each family, to identify potentially pathogenic variants. PCR and Sanger sequencing of the *CEP290* gene fragment encompassing a position c.2991+1665A>G in the intron 26, which is the most common variant in this gene, was previously conducted in all of these patients, who were subjected to WES. DNA samples of 15 probands from 15 families were subjected to exome capture and high-throughput sequencing. Sequencing libraries were prepared with Twist Human Core Kit (Twist Bioscience). Sequencing of 100 bp paired-ends reads was performed on NovaSeq 6000. The human reference genome (hg19) was used. The variants in 25 genes that are known to be implicated in the pathogenesis of LCA were filtered and analyzed. The following LCA-related genes were screened for potentially pathogenic variants: *AIPL1*, *CABP4*, *CCT2*, *CEP290*, *CLUAP1*, *CRB1*, *CRX*, *DTHD1*, *GDF6*, *GUCY2D*, *IFT140*, *IMPDH1*, *IQCB1*, *KCNJ13*, *LCA5*, *LRAT*, *NMNAT1*, *OTX2*, *PRPH2*, *RD3*, *RDH12*, *RPE65*, *RPGRIP1*, *SPATA7*, and *TULP1.* Moreover, in two patients, in whom no potentially pathogenic variants were identified in LCA genes, 294 genes (Supplementary material 1a) associated with IRDs were screened for mutations.

The targeted NGS of 275 IRD genes (Supplementary material 1b) was performed in 12 index patients from 12 families, using the SeqCap EZ HyperCap protocol and the NimbleGene SeqCap EZ probe set (Roche) on a NextSeq 500 Illumina sequencing system. The panel analysis included a variant c.2991+1665A>G in the intron 26 of the *CEP290* gene.

Variants identified with the WES and NGS panel of IRD genes were cross-checked to the Leiden Open Variation Database (LOVD) (https://www.lovd.nl/), Human Gene Mutation Database (HGMD) (https://www.hgmd.cf.ac.uk/ac/index.php), ClinVar (https://www.ncbi.nlm.nih.gov/clinvar/), dbSNP (https://www.ncbi.nlm.nih.gov/snp/), and GnomAD browser (Genome Aggregation Database) (https://gnomad.broadinstitute.org/). Variants were visualized by use of an Integrative Genomics Viewer (IGV; Broad Institute and the Regents of the University of California). CADD (Combined Annotation Dependent Depletion) (https://cadd.gs.washington.edu/) and Fathmm (Functional Analysis Through Hidden Markov Models) (http://fathmm.biocompute.org.uk/) were additionally used to predict the possible effect of two novel splicing variants.

Variants were annotated against the reference sequences of the analyzed genes (see the captions under Table 2) following the HGVS (Human Genome Variation Society) nomenclature (http://varnomen.hgvs.org/)*.* The targeted PCR followed by Sanger sequencing was applied to confirm their presence in all index patients and to study their familial segregation. Segregation analysis was conducted in 21 out of 27 families including all families carrying novel variants (10 families) and 12 families with known variants. For primer sequences, see Supplementary Table 1. The sequencing products were separated on an ABI 3130xl capillary sequencer (Applied Biosystems).

**Table 2:** Variants in LCA-associated genes identified in 27 Polish families

Family no.	Mode of inheritance	Gene^1^	Causative and coexisting variants				Pathogenicity prediction in protein level			Classification according to ACMG^3^	Allele frequency (gnomAD browser)^4^	Molecular method of searching the variants^5^
			Nucleotide	Exon/intron no.	Protein	Status	SIFT	PROVEAN	PolyPhen-2^2^			
23	AR	*CEP290*	c.1753C>T	e.18	p.Q585*	Het	**-**	**-**	**-**	P	None	WES
			c.2991+1655A>G	i.26	p.C998*	Het	**-**	**-**	**-**	P	None	NGS panel
40												NGS panel
46												
24	AR	*LCA5*	**c.1555_1558del**	e.8	p.F519Mfs*73	Hom	**-**	**-**	**-**	LP	None	WES
25	AR	*GUCY2D*	c.2302C>T	e.12	p.R768W	Het	Damaging	Deleterious	P. damaging	P	0.000151	WES
			c.2598G>C	e.14	p.K866N	Het	Damaging	Deleterious	P. damaging	VUS	0.0000065	
		*CEP290*	c.4577A>T	e.35	p.E1526V	Het	Damaging	Neutral	P. damaging	VUS	0.0000241	
26	AD	*CRX*	c.585C>A	e.4	p.Y195*	Het	**-**	**-**	**-**	P	None	WES
27	AR	*CRB1*	**c.1457T>C**	e.6	**p.L486P**	Het	Damaging	Deleterious	P. damaging	LP	None	WES
			c.2843G>A	e.9	p.C948Y	He	Damaging	Deleterious	P. damaging	P	0.000212	
28	AR	*CEP290*	c.289G>T	e.5	p.E97*	Het	**-**	**-**	**-**	P	0.0000226	WES
			c.2991+1655A>G	i.26	p.C998*	Het	**-**	**-**	**-**	P	None	
29	AR	*RPGRIP1*	c.2465_2468dup	e.17	p.A824Ifs*11	Hom	**-**	**-**	**-**	P	0.0000040	WES
30	AR	*NMNAT1*	**c.292G>C**	e.3	**p.V98L**	Hom	Damaging	Deleterious	Benign	LP	None	WES
		*GUCY2D*	c.2179G>A	e.11	p.G727S	Het	Damaging	Deleterious	P. damaging	LP	0.000363	
31	AR	*GUCY2D*	**c.566_571del**	e.2	p.A189Vfs*131	Hom	**-**	**-**	**-**	LP	None	WES
			**insTGGGTGGAGG**									
32	AR	*GUCY2D*	c.2291del	e.12	p.P764Lfs*20	Hom	**-**	**-**	**-**	P	0.0000039	WES
33	AR	*NMNAT1*	c.769G>A	e.5	p.E257L	Het	Tolerated	Neutral	Bening	LP	0.000702	WES
			**NC_000001.11** (**9970990_978973)del**			Het	**-**	**-**	**-**	P	None	
34	AR	*CRB1*	c.2042G>A	e.6	p.C681Y	Het	Damaging	Deleterious	P. damaging	P	0.0000039	WES
			**c.3474T>A**	e.9	p.Y1158*	Het	**-**	**-**	**-**	LP	None	
35	AR	*CEP290*	c.2991+1655A>G	i.26	p.C998*	Het	**-**	**-**	**-**	P	None	WES
			c.6277delG	e.46	p.V2093Sfs*4	Het	**-**	**-**	**-**	P	0.0000467	
36	AR	*CEP290*	c.1078C>T	e.13	p.R360*	Het	**-**	**-**	**-**	P	0.0000264	WES
			c.2991+1655A>G	i.26	p.C998*	Het	**-**	**-**	**-**	P	None	
37	AR	*LRAT*	c.139C>T	e.2	p.R47*	Hom	**-**	**-**	**-**	P	0.0000119	NGS panel
38	AR	*CRB1*	**c.1660del**	e.6	p.V554Cfs*19	Het	**-**	**-**	**-**	P	None	NGS panel
			c.2042G>A	e.6	p.C681Y	Het	Damaging	Deleterious	P. damaging	P	0.0000039	
39	AR	*CRB1*	c.2843G>A	e.9	p.C948Y	Hom	Damaging	Deleterious	P. damaging	P	0.000212	NGS panel
41	AR	*CRB1*	**c.1342C>T**	e.6	p.Q448*	Het	**-**	**-**	**-**	P	None	NGS panel
			c.2843G>A	e.9	p.C948Y	Het	Damaging	Deleterious	P. damaging	P	0.000212	
42	AR	*CEP290*	**c.1522+2T>C**^**a**^	i.15	p.?	Het	**-**	**-**	**-**	P	None	WES
			**c.5012+1G>A**^**b**^	i.37	p.?	Het	**-**	**-**	**-**	P	None	
43	AR	*CRB1*	c.2042G>A	e.6	p.C681Y	Het	Damaging	Deleterious	P. damaging	P	0.0000039	NGS panel
			c.2843G>A	e.9	p.C948Y	Het	Damaging	Deleterious	P. damaging	P	0.000212	
44	AR	*NMNAT1*	c.769G>A	e.5	p.E257L	Het	Tolerated	Neutral	Bening	LP	0.000702	NGS panel
			**c.839A>T**	e.5	p.(*280Lext*15)	Het	**-**	**-**	**-**	LP	None	
45	AR	*CEP290*	c.2991+1655A>G	i.26	p.C998*	Hom	**-**	**-**	**-**	P	None	NGS panel
		*RPGRIP1*	c.1216del	e.11	p.L406Yfs*36	Het	**-**	**-**	**-**	LP	None	
47	AR	*RPGRIP1*	c.2236G>A	e.16	p.(G746R)	Het	Damaging	Deleterious	P. damaging	LP	0.0000122	NGS panel
			c.2367+1G>A	i.16	p.?	Het	**-**	**-**	**-**	P	None	
48	AR	*CEP290*	c.2991+1655A>G	i.26	p.C998*	Het	**-**	**-**	**-**	P	None	NGS panel
			c.5587-1G>C	i.40	p.?	Het	**-**	**-**	**-**	P	0.0000143	
49	AR	*CEP290*	c.2991+1655A>G	i.26	p.C998*	Het	**-**	**-**	**-**	P	None	NGS panel
			c.4882C>T	e.37	p.Q1628*	Het	**-**	**-**	**-**	P	0.0000369	

Novel variants are marked in bold. The hyphen means that the prediction in protein level was not performed for the variant (nor necessary or improper for this variant)

^1^The reference sequences of the genes, in which the variants were detected, were as follows: *CEP290* NM_025114.4, *CRB1* NM_201253.2, *GUCY2D* NM_000180.4, *NMNAT1* NM_002431.4, *RPGRIP1* NM_020366, *CRX* NM_000554.6, *LRAT1* NM_004744.3, *LCA5* NM_001122769.3, and *IQCB1* NM_001023570.4

^2^PolyPhen2: *P. damaging* probably damaging

^3^Classification according to ACMG: *P* pathogenic, *LP* likely pathogenic, *VUS* uncertain significance

^4^Allele frequency is listed according to GnomAD (Genome Aggregation Database), accessed 18 February 2022

^a, b^Splicing variants submitted to additional potential pathogenicity prediction in protein level analyses with the use of CADD and Fathmm software. The results of the analyses revealed that both variants are deleterious. For the variant c.1522+2T>C in the *CEP290* gene, the CADD score is 33 and the Fathmm score is 0.99. For the variant c.5012+1G>A in the *CEP290* gene, the CADD score is 25.5 and the Fathmm score is 0.99

^5^WES was conducted in patients diagnosed with LCA from May 2020 to April 2021, while NGS retinal panel was applied in these patients, who were referred to the genetic clinic before May 2020 and after April 2021

Quantitative PCR (qPCR) was applied to confirm the heterozygous deletion identified with WES analysis in the *NMNAT1* gene, in female patient no. 33-110, as well as to perform co-segregation analysis in the family and narrow down the deletion coordinates. We used SYBR dye-based master mix (SYBR Green PCR Master Mix; ThermoFisher Scientific) and ran the reactions on the ViiA™ 7 Real-Time thermal cycler (ThermoFisher Scientific) as described previously (Sowińska-Seidler et al. 2018). Reaction conditions are available upon request. For primer sequences, see Supplementary Table 2.

Comparative genomic hybridization to arrays (aCGH) was performed in patient 35-119, who presented delayed psychomotor development and dysmorphic features. The aCGH was conducted with the use of Sure Print G3 CGH ISCA v2, 8×60k (Agilent Technologies) following the standard protocol provided by the manufacturer. Analysis was carried out with CytoGenomics software (Agilent Technologies) using the following settings: window size 0.10 Mb, filter—5 probes, DLR spread <0.3.

## Results

### Clinical observations

**Fig. 1:**
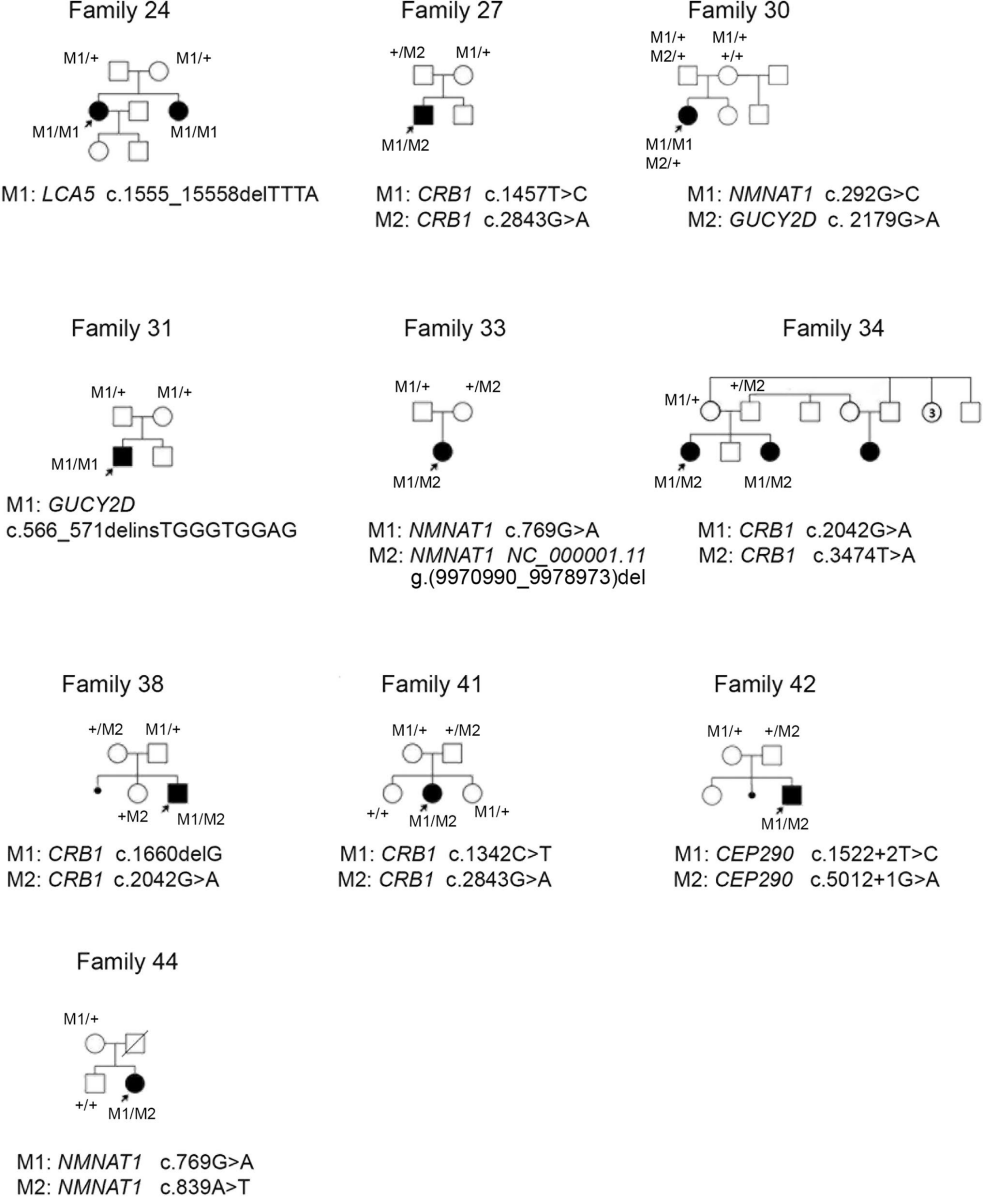
Pedigrees of the families with novel variants in LCA genes. The dark-filled symbols indicate individuals affected with LCA and unfilled symbols point out unaffected individuals. The slash represents a deceased person. The arrows mark probands

We examined 31 patients from 27 families, aged 1–55 years, with a clinical diagnosis of LCA. In 26 families, the pedigrees analyses suggested an autosomal recessive mode of inheritance, while in one family the disease appears to have a dominant inheritance pattern. Pedigrees of the examined families together with the results of segregation analysis are shown in Fig. 1 (families with novel variants) and Supplementary Fig. 1 (families with known variants). Most of the patients exhibited typical LCA symptoms, while two boys (35-119 and 42-158) presented some extra-ocular features. Clinical symptoms and the results of the ophthalmologic examination of the probands are summarized in Table 1.

All but one patient (woman no. 47-164) presented nystagmus as one of the first symptoms, and most of them revealed this clinical feature shortly after birth or within the first 2 months. Fundoscopic imaging revealed peripheral or also macular pigment deposits in most patients’ retinas. In four children aged 1–2 years (patients 36-133, 45-161, 46-163, and 49-167), the fundus appearance was normal. In a 33-year-old woman (patient 24-72), funduscopy was performed just once when she was a 2-month-old baby and revealed a pale optic disc. In patient no. 28-89, who is now 9 years old, funduscopy was performed at the age of 6 months and the fundus appearance was normal. In patient no. 23-66, the fundus image was blurred due to a cataract. Electroretinography was performed in 24 out of 31 patients (excluding patients 26-80, 26-81, 28-89, 29-92, 37-144, 38-146, and 45-161). In most patients, the scotopic and photopic responses were extinguished.

### Molecular analyses results

**Fig. 2:**
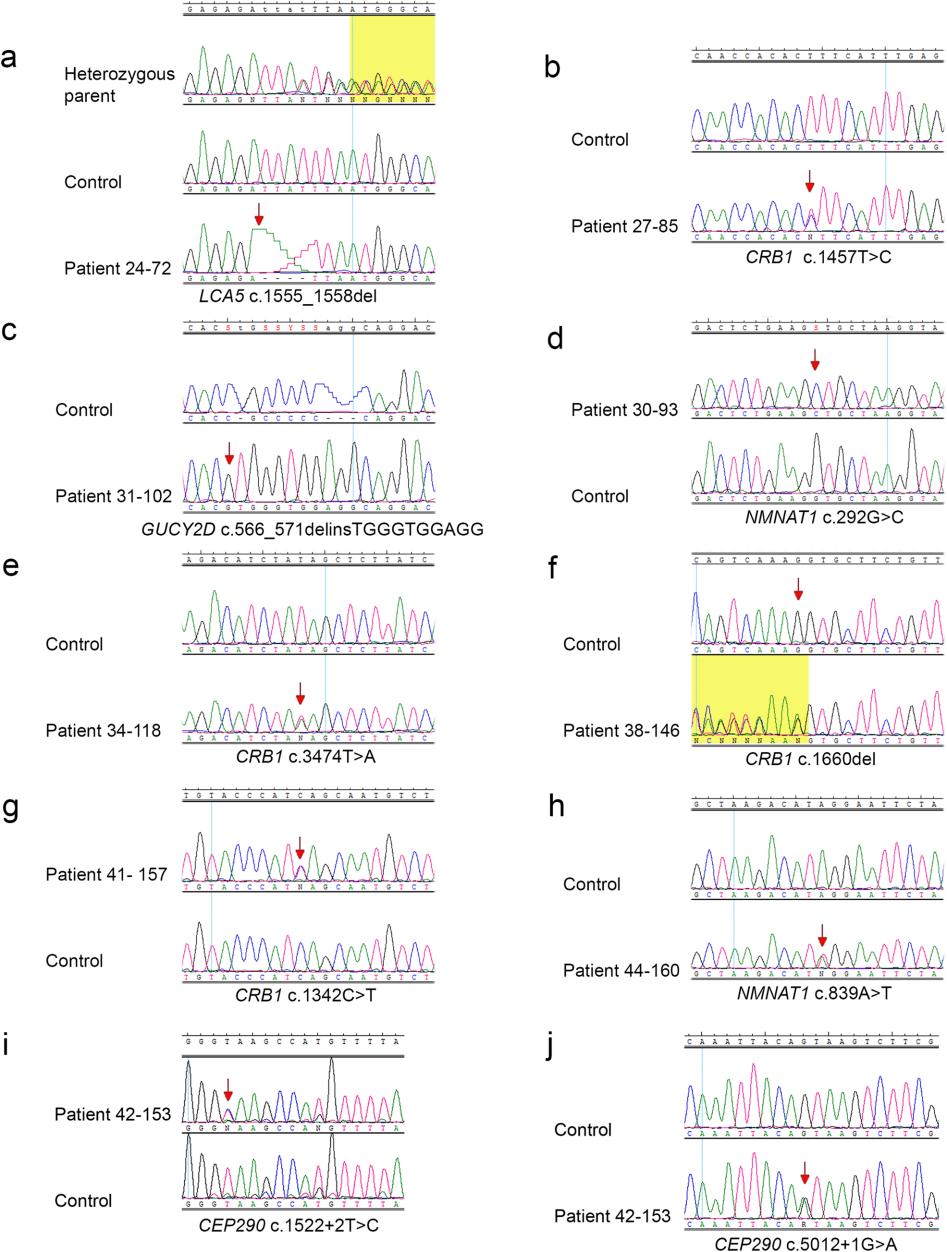
Chromatograms showing novel variants identified in LCA genes. The arrows indicate nucleotides that have been changed or the first nucleotides involved in variants. The yellow background appears in two chromatograms showing frameshift variants (**a** and **f**)

The results of WES analysis performed in 15 patients and targeted NGS panel in 12 patients revealed 28 potentially pathogenic variants including 11 novel, in 8 genes: *CEP290*, *CRB1*, *GUCY2D*, *NMNAT1*, *RPGRIP1*, *CRX*, *LRAT1*, and *LCA5*. None of the novel variants was reported in GnomAD, LOVD, HGMD, dbSNP, and ClinVar variant databases. All the identified variants in LCA genes together with their frequency in GnomAD and predictions of pathogenicity are shown in Table 2. The chromatograms of novel variants are shown in Fig. 2. The results of the segregation analysis were consistent with the expected mode of inheritance in all the examined families.

In 10 out of 27 examined families, we detected potentially pathogenic variants in the *CEP290* gene, which allowed us to make a diagnosis of type 10 Leber congenital amaurosis. Altogether, 8 variants were identified in the *CEP290* gene including two novel. These novel variants were detected in patient 42-158 presenting a progressive deterioration of visual acuity, as well as developmental disorder and motor hyperactivity. The patient was previously suspected to suffer from Batten disease, but this form of neuronal ceroid lipofuscinosis was excluded based on WES analysis. Two years later, the reanalysis of WES results revealed two novel intronic variants in the *CEP290* gene, which led to the diagnosis of LCA. Both of the variants identified in this boy: c.1522+2T>C and c.5012+1G>A are potentially pathogenic as they affect the donor splice sites. We inspected these variants using two in silico prediction software: CADD and Fathmm, which confirmed that both of them are deleterious (see the scores in captions under Table 2).

The intronic variant c.2991+1655A>G (p.C998*) in the *CEP290* gene was identified in 9 families (no. 23-66, 28-88, 35-119, 36-133, 40-156, 45-161, 46-163,48-166, and 49-167).

Interestingly, two *CEP290* gene variants: the intronic substitution c.2991+1655A>G and the deletion c.6277delG (p.V2093Sfs*4), were detected in patient 35-119, presenting an extra-ocular phenotype. The previously performed aCGH analysis revealed a microduplication MidXq28 of 256 kb (chrX:153576890-153832724) that contributes to the dysmorphic features and developmental delay but does not explain the severe retinal dystrophy observed in the boy, therefore, we conducted WES.

The diagnosis of LCA8 was established based on NGS results that revealed *CRB1* gene variants in 6 families: 27, 34, 38, 39, 41, and 43. Among 6 detected variants, 4 were novel including 2 nonsense: c.3474T>A (p.Y1158*) and c.1342C>T (p.Q448*), a frameshift: c.1660del (p.V554Cfs*19), and a missense variant c.1457T>C (p.L486P). Visual acuity in examined patients with *CRB1* mutations ranged from 1/10 in a 14-year-old boy carrying missense variants: c.2042G>A and c.2843G>A (patient 43-159), to light perception in two sisters, aged 29- and 21-year-old (family 34) carrying a novel nonsense variant c.3474T>A and missense substitution c.2042G>A.

In 3 families, the WES analysis results allowed for making a diagnosis of LCA1. Among the 4 *GUCY2D* variants detected in our study group, one was novel: c.566_571delinsTGGGTGGAGG. This deletion-insertion causes a frameshift and introduces a premature stop codon (p.A189Vfs*131). This likely pathogenic homozygous variant was detected in a 23-year-old man with poor light perception (no. 31-102). The segregation analysis revealed that this frameshift variant was inherited from both his unaffected, heterozygous parents, who are probably consanguineous, as their grandparents came from one small village.

NGS techniques revealed 4 *NMNAT1* variants in 3 out of 27 examined families. Three variants were novel including two missense substitutions and a copy number variation (CNV). The novel substitution c.292G>C (p.V98L) was identified in a homozygous state in a 21-year-old woman (no. 30-93), with visual acuity restricted to poor light perception. The in silico predictions of the variant’s potential pathogenicity with the use of SIFT and PROVEAN, indicated that the substitution is damaging, while based on Polyphen2 predictions, the variant is supposed to be benign. According to the ACMG classification, the substitution is likely pathogenic. Moreover, in this female patient, WES analysis revealed the presence of heterozygous *GUCY2D* substitution: c.2179G>A (p.G727S), classified as likely pathogenic based on ACMG classification. The *NMNAT1* novel variant, as well as the *GUCY2D* substitution segregated with the disease.

**Fig. 3:**
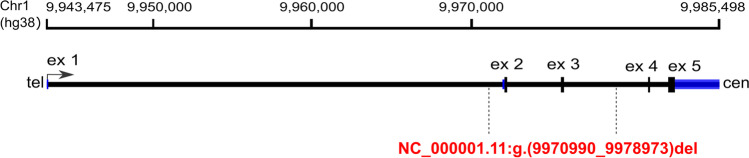
Schematic representation of the *NMNAT1* gene. The deleted region is indicated by vertical dashed lines. Blue bars indicate 5′ and 3′ UTRs; ex, exons

The analysis targeting copy number variants (CNVs) using WES data allowed for the identification of a deletion encompassing exons 2–3 in the *NMNAT1* gene*.* The variant was detected in a form of a compound heterozygote in a 10-year-old girl with a severe form of LCA. A recurrent variant c.769G>A was identified on the second allele. The girl’s DNA was subjected to molecular analyses a few years ago with the use of an NGS panel of IRD genes and WES analysis at two different diagnostic centers. No causative variants were detected at that time. Segregation analysis performed using qPCR revealed that the deletion identified with WES was inherited from the healthy mother of the proband (Supplementary Fig. 2). The assay allowed us to narrow down the deletion coordinates (NC_000001.11 g.(9970990_9978973)del) and to establish the approximate size of the CNV to be 7984 bp (Fig. 3).

The novel *NMNAT1* substitution c.839A>T (p.(*280Lext*15)) was detected in a compound heterozygous state with a c.769G>A variant in-trans, in the 7-year-old girl with (no. 44-160). The girl’s visual acuity is 0.02, and she has hyperopic astigmatism. The novel transversion affects the last amino acid position causing elongation of the protein chain by 15 amino acids, and it is classified as likely pathogenic based on ACMG classification.

In one family (no. 24), WES analysis revealed the presence of a novel, homozygous *LCA5* gene variant. The deletion: c.1555_1558del (p.F519Mfs*73) was detected in the blind female patient (no. 24-72). The variant causes a frameshift and introduces a premature stop codon, which results in the formation of the protein abbreviated by 105 amino acids. The same but heterozygous deletion was detected in both parents of the proband, although there is no information regarding their consanguinity.

Aside from 11 novel variants in the LCA genes, we detected numerous rare variants. Two rare variants were identified in the *GUCY2D* gene: a homozygous deletion c.2291delC (p.P764Lfs*20) in patient 32-107, and the substitution c.2598G>C (p.K866N) identified in a form of compound heterozygote with a known variant c.2302C>T, in two sisters from the family 25. The c.2598G>C variant was predicted to be damaging with the use of SIFT, Provean, and probably damaging with the use of PolyPhen2; nevertheless, according to ACMG classification, it was classified as a variant of uncertain significance (VUS). However, this extremely rare variant was previously detected in a compound heterozygote form in a patient carrying also the c.2302C>T substitution and diagnosed with retinitis pigmentosa. The c.2598G>C substitution is located at a highly conserved position (Coppieters et al. 2010). Furthermore, in family 25, we detected also a heterozygous substitution c.4577A>T (p.E1526V) in the *CEP290* gene, which is also a rare variant classified as VUS. Both variants in *GUCY2D*, as well as the *CEP290* variant segregated within the family.

The extremely rare variant c.585C>A introducing premature stop codon (p.Y195*) was detected in the *CRX* gene in the family 26 presenting an AD mode of inheritance. This nonsense variant was not reported in GnomAD, nor LOVD and HGMD. To our best knowledge, it was only reported once, in two individuals (Stone 2007).

## Discussion

In this report, we summarized the results of molecular analysis based on two NGS techniques: targeted NGS of 275 IRD genes and WES analysis, applied in 27 families clinically diagnosed with Leber Congenital Amaurosis. We identified 29 potentially pathogenic variants, including 11 novel, in 8 LCA genes: *CEP290*, *CRB1*, *GUCY2D*, *NMNAT1*, *RPGRIP1*, *CRX*, *LRAT1*, and *LCA5*.

The results of this study support our previous findings (Skorczyk-Werner et al. 2020) that the *CEP290* gene variants are the most common cause of LCA in Polish patients. To sum up, among 49 families (22 families described in our previous report and 27 families characterized in this study), the *CEP290* gene variants were detected in 18 families (10 reported in this study and 8 previously described), which accounts for almost 37%. According to the literature, *CEP290* gene biallelic variants are the most common cause of LCA in Caucasians and account for 15–30% of all cases (den Hollander et al. 2006; Kumaran et al. 2017; Leroy et al. 2021). Therefore, we can conclude that in Polish patients LCA10 is even more common than in the other populations.

The intronic substitution c.2991+1655A>G (p.C998*) is the most common variant in Polish LCA patients since it was detected in 16 out of 49 families, which accounts for 35% of all cases. The variant was identified in 16 out of 18 LCA10 families, which is almost 89% of families with *CEP290* variants.

In this study, we also identified causative variants in the *CEP290* gene in two patients with extra-ocular symptoms: two novel intronic variants in patient 42-158 and known variants in patient 35-119 carrying also a microduplication MidXq28 (see Tables 1 and 2). To our best knowledge, here we report the first case of a patient with the coexistence of MidXq28 syndrome and LCA10. Based on these two patients’ clinical history, we conclude that in every case of significantly reduced visual acuity and nystagmus in infancy and early childhood, even when other neurological symptoms are present, LCA should be suspected.

The vision is usually severely impaired in patients with LCA10. However, we identified the known *CEP290* variants in one patient, a 10-year-old girl (48-166) with almost normal visual acuity with periodic decline. The girl harbors the recurrent c.2991+1655A>G and the potentially pathogenic rare substitution c.5587-1G>C.

Interestingly, a rare *CEP290* gene variant c.1753C>T (p.Q585*) was detected in a form of a compound heterozygote together with the c.2991+1655A>G variant in three unrelated families (no. 23, 40, and 46). This substitution was not reported in the GnomAD browser, as well as LOVD, HGMD, and dbSNP, but it has been described in the literature (den Hollander et al. 2006; Stone et al. 2017) and ClinVar database. Therefore, we can assume that this variant is more common in Polish LCA patients than in patients of other nationalities.

One more *CEP290* gene variant: the known substitution c.4882C>T (p.Q1628*) (Feldhaus et al. 2020), detected in patient 49-167, was also identified in 3 families reported in our previous study (Skorczyk-Werner et al. 2020).

Based on the results of this study, *CRB1* gene variants are the second cause of LCA in Polish patients, since we detected six variants, including four novel. Taking together six families affected with LCA8 described in this study and two families with the *CRB1* variants reported in our previous study (Skorczyk-Werner et al. 2020), this form of the disease was diagnosed in 8 out of 49 Polish families, which accounts for 16%. According to the literature, approximately 9–17% of LCA cases are related to *CRB1* mutations (Kumaran et al. 2017; Wang et al. 2015; Huang et al. 2021). Therefore, based on our results, the frequency of LCA8 among Polish patients is relatively high, similar to those observed in the Chinese population (Kumaran et al. 2017; Wang et al. 2015; Huang et al. 2021).

The most common *CRB1* variant in the group of Polish patients appeared to be the substitution c.2843G>A, which was detected altogether in 6 out of 8 families described in this study and in our previous report (Skorczyk-Werner et al. 2020). Thus, this substitution is the second most frequent variant in Polish LCA patients. Another *CRB1* variant that can be considered a common one in Polish patients is the c.2042G>A (p.C681Y) identified in 3 families.

The phenotype-genotype correlations in our cohort of patients with *CRB1* variants were challenging to perform due to a broad age range of patients (6–49 years old). In general, visual acuity was better in younger patients as compared to older individuals. Nevertheless, the most severe form of visual disability was observed in two sisters, in their twenties, harboring a novel variant c.3474T>A and a known substitution c.2042G>A. All patients with *CRB1* variants had nystagmus as a first symptom and most of them presented photophobia as well as night blindness. All but two patients presented high hyperopia (see Table 1), which is typical for LCA8.

Based on the results of this study and our previous report, the third most common cause of LCA in Polish patients are *GUCY2D* gene variants. Altogether, in a group of 49 patients with LCA confirmed by molecular analyses, we detected nine *GUCY2D* variants in seven families, including three novel, as well as two rare variants. Thus, LCA1 accounts for 14% of cases, which is consistent with the literature data indicating that the *GUCY2D* gene mutations are detected in 6–21% of patients (Huang et al. 2021).

The results of our study on Polish patients confirm the statement that LCA1 is a severe form of the disease, causing early profound visual loss (Kumaran et al. 2017), as we observed poor visual acuity, restricted to light perception in most patients carrying biallelic *GUCY2D* variants, even in a few years old children. Better visual acuity was observed only in two sisters from the family 25 carrying c.2302C>T and c.2598G>C variants and in two children affected with c.2302C>T and c.721+2T>C substitutions described in our previous publication (Skorczyk-Werner et al. 2020). The c.2302C>T substitution appeared to be the most common *GUCY2D* variant in our group of patients, being detected in 3 out of 7 families.

LCA9 was diagnosed in 5 out of 49 families, which accounts for 10% of cases. The frequency of *NMNAT1* variants in our patients can be considered as high since it was reported that LCA9 is diagnosed in about 4–8% of cases (Falk et al. 2012; Perrault et al. 2012; Yi et al. 2021). Three variants detected in this study were novel: c.292G>C (p.V98L), c.839A>T (p.(*280Lext*15)), and the a 7984 bp deletion.

The substitution p.V98L identified in a homozygous state in patient 30-93 was evaluated as likely pathogenic according to ACMG classification (PM2, PM5, PP2, PP3); however, based on Polyphen2 analysis, it is predicted to be benign. Both valine and glycine are hydrophobic amino acids with aliphatic R-chains. Nevertheless, the analysis employing the mCSM tool (http://biosig.unimelb.edu.au/mcsm/) revealed that the predicted stability change (ΔΔG) was equal to −0.669kcal/mol, indicating that the variant was evaluated as destabilizing. Moreover, the pathogenic substitution of valine at the same amino acid position to glycine was previously reported (c.293T>G; p.V98G), and it was suspected to disturb local interactions, which in consequence may alter the active site of the NMNAT1 enzyme affecting the enzymatic activity (Chiang et al. 2012). Additionally, in this patient (no. 30-93), WES analysis revealed the presence of heterozygous *GUCY2D* substitution: c.2179G>A (p.G727S), which is classified as likely pathogenic (PM2, PM1, PP3) based on ACMG classification. The variant was reported in two cases of retinitis pigmentosa (RP) together with causative variants in other RP-associated genes. The c.2179G>A variant was finally evaluated to be a non-disease-causing that likely does not contribute to the patients’ disease (Verdina et al. 2021; Costa et al. 2017). According to the STRING database (https://string-db.org/), the *GUCY2D* gene is not a predicted functional partner of *NMNAT1*, but we cannot exclude the c.2179G>A variant as a modifying factor of the disease.

Another novel *NMNAT1* variant: the fail-to-stop mutation c.839A>T (p.(*280Lext*15)) was identified based on NGS of IRD genes panel analysis in a form of a compound heterozygote together with c.769G>A variant, in the 7-year-old girl (no. 44-160). Interestingly, although LCA9 is a severe form of the disease, the visual acuity of this girl was relatively good; however, the funduscopic imaging shows numerous central and peripheral retinal pigment deposits. The p.(*280Lext*15) variant is predicted to be likely pathogenic (PM4, PM2, BP4); however, we can assume that it can cause a milder phenotype than e.g., nonsense mutations.

The c.769G>A (p.E257L) substitution, which is the most frequently encountered *NMNAT1* variant, reported in more than 70% of patients affected with LCA9 (Chiang et al. 2012; Falk et al. 2012; Perrault et al. 2012; Sasaki et al. 2015; Thompson et al. 2017) was detected in four out of five Polish families with LCA9.

In the described group of 27 LCA families, we also identified numerous extremely rare or rare variants, which may appear to be more frequent in the Polish population, in studies on a larger group of patients.

In this study, we did not identify any causative variants in the *RPE65* gene, even though we have previously detected 4 variants in 3 families, and thus, we reported mutations in this gene as the third most common cause of LCA (Skorczyk-Werner et al. 2020). Based on the results of this study and our previous report, the LCA2 was diagnosed only in 2% of our patients.

Both targeted NGS and WES analyses allowed us to successfully determine the molecular background of LCA in all 27 studied families. Almost 96% of our patients diagnosed based on NGS techniques received a full molecular diagnosis, including 27 families reported in this study, 13 families subjected to NGS panel for LCA genes (Skorczyk-Werner et al. 2020), and 2 families without a molecular diagnosis made with WES.

Furthermore, the development of NGS technology has allowed for the detection of CNVs, which were overlooked through previous versions of NGS-based assays. This enabled us to identify a deletion in the *NMNAT1* gene in patient 33-110. The deletion was undetected with the use of the NGS panel and WES analysis performed a few years earlier in different laboratories.

Due to the recent therapeutic approaches in LCA, searching for the molecular background of this previously uncurable disease takes on greater significance. Since the Luxturna therapy, which is a subretinal surgical delivery of live non-replication adenoviral vector carrying *RPE65* gene (Voretigene Neparvovec-rzyl—brand name: Luxturna^TM^) (Russell et al. 2017) is available in Poland, it seems that there is a growing interest in performing genetic testing to understand the underlying cause of the LCA, as well as other IRDs, among Polish patients.

Clinical trials are underway also in patients affected with mutations in *CEP290* and *GUCY2D* genes. A great hope for treatment for Polish patients are especially clinical trials focusing on c.2991+1655A>G mutation in the *CEP290* gene, as more than 35% of them are affected by this intronic variant. Two different approaches to treating the form of LCA caused by this mutation are in their clinical trials. Moreover, research on animal models to develop therapies caused by mutations in other genes, i.e., *AIPL1*, *RPGRIP1*, *LCA5*, and *RDH12* is also promising.

To sum up, we demonstrated the clinical utility of both the NGS IRDs panel and WES with the analysis of 25 LCA gene approaches in searching for a molecular background of LCA. We identified potentially pathogenic mutations in 8 genes including 11 novel mutations in the Polish cohort of patients that broaden the spectrum of LCA gene mutations. Most variants were detected in the *CEP290*, *CRB1*, *GUCY2D*, and *NMNAT1* genes. Two variants appeared to be the most commonly detected in Polish patients: the intronic substitution c.2991+1655A>G in the *CEP290* gene and c.2843G>A in the *CRB1* gene. Although there are no clear phenotype-genotype correlations in our group of patients, we indicate phenotypic variability between LCA forms and between the effects of different types of mutations. We have observed a milder course of the disease for some genetic variants, which may be an opportunity for young patients to be treated with gene therapy.

## Supplementary information

Supplementary Fig. 1 (PNG 560 kb)

High resolution image (TIF 2621 kb)

Supplementary Fig. 2 (PNG 252 kb)

High resolution image (TIF 1585 kb)

ESM 1 (DOCX 15 kb)

Supplementary Table 1 (DOCX 19 kb)

Supplementary Table 2 (DOCX 18 kb)
