# Supplementary figures and images for: Molecular background of Leber congenital amaurosis in a Polish cohort of patients—novel variants discovered by NGS
Source: J Appl Genet. 2022 Nov 12;64(1):89–104. doi: 10.1007/s13353-022-00733-9 (PMC9837007; doi:10.1007/s13353-022-00733-9)

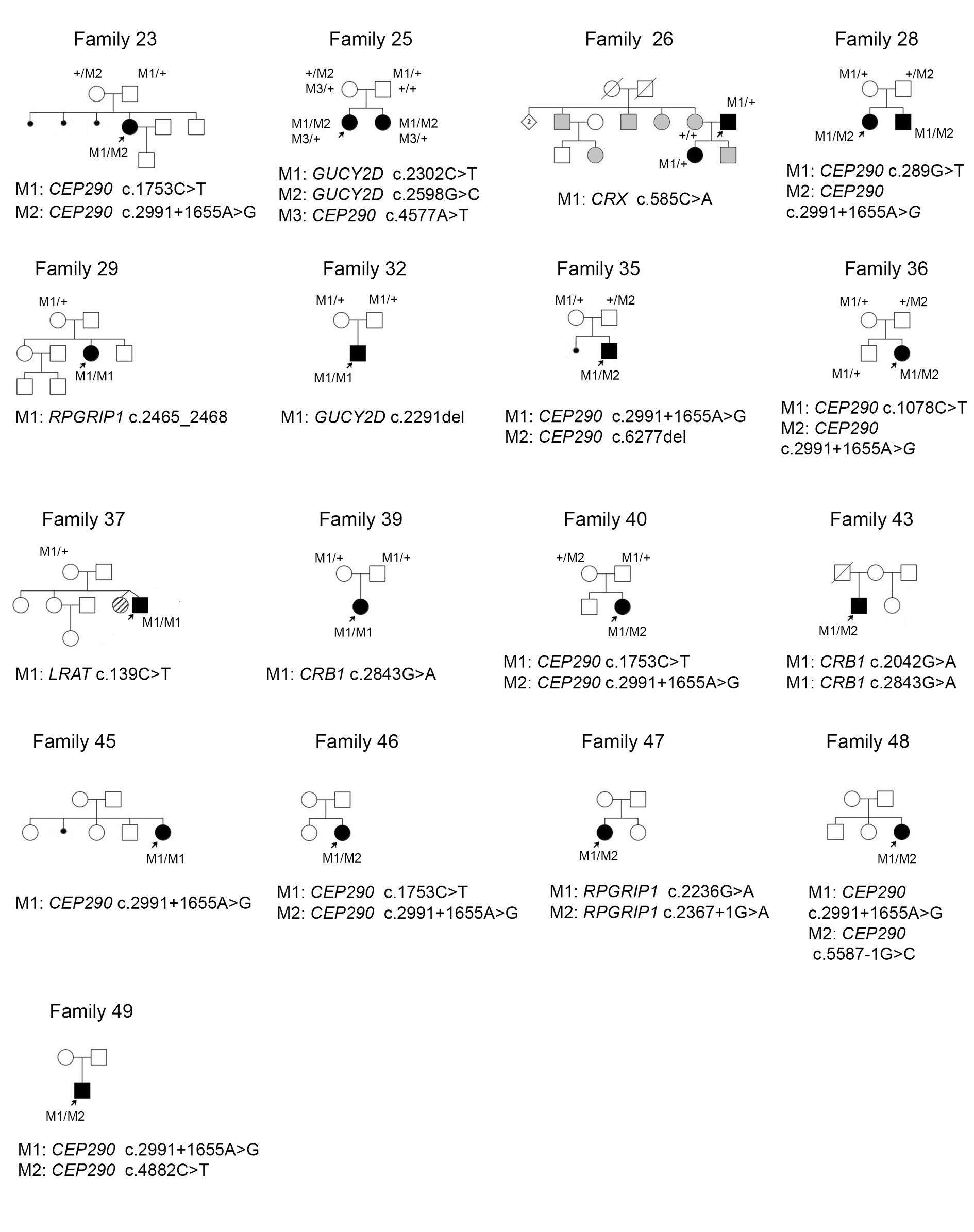

Supplement: Supplementary file 1 — (PNG 560 kb) [file 13353_2022_733_Fig4_ESM.png]

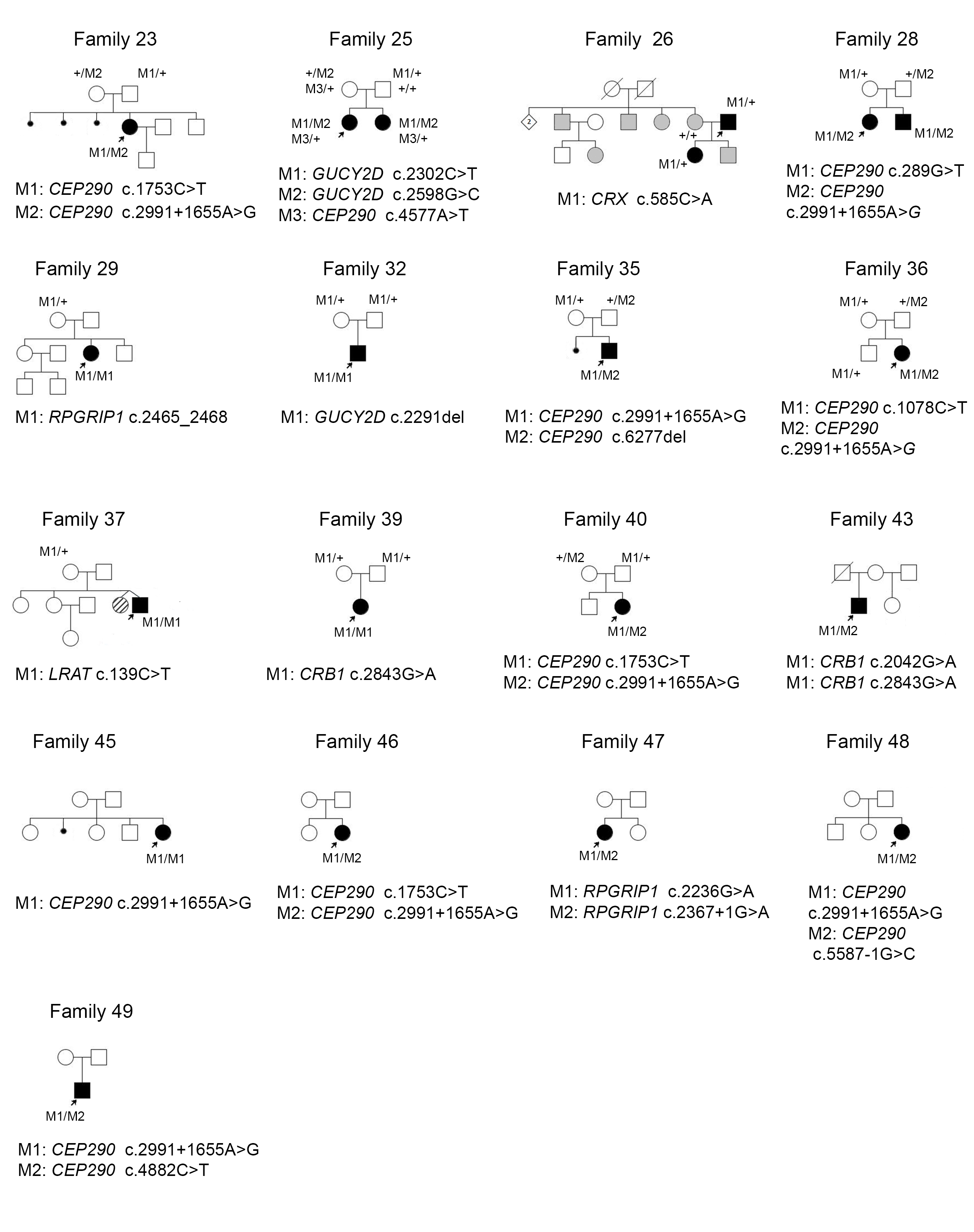

Supplement: Supplementary file 2 — High resolution image (TIF 2621 kb) [file 13353_2022_733_MOESM1_ESM.tif]

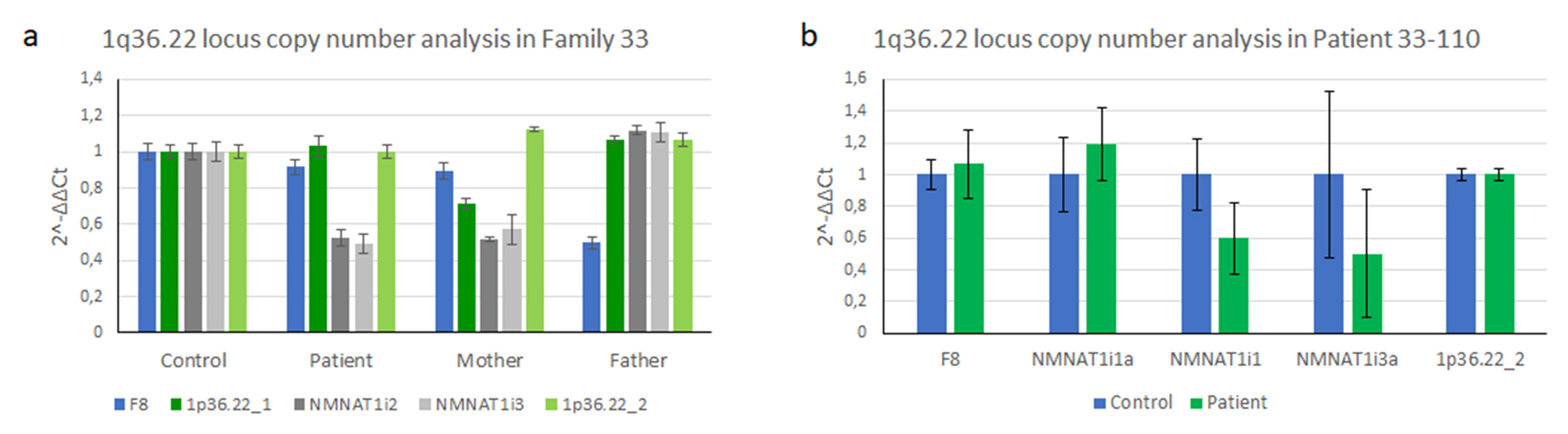

Supplement: Supplementary file 3 — (PNG 252 kb) [file 13353_2022_733_Fig5_ESM.png]

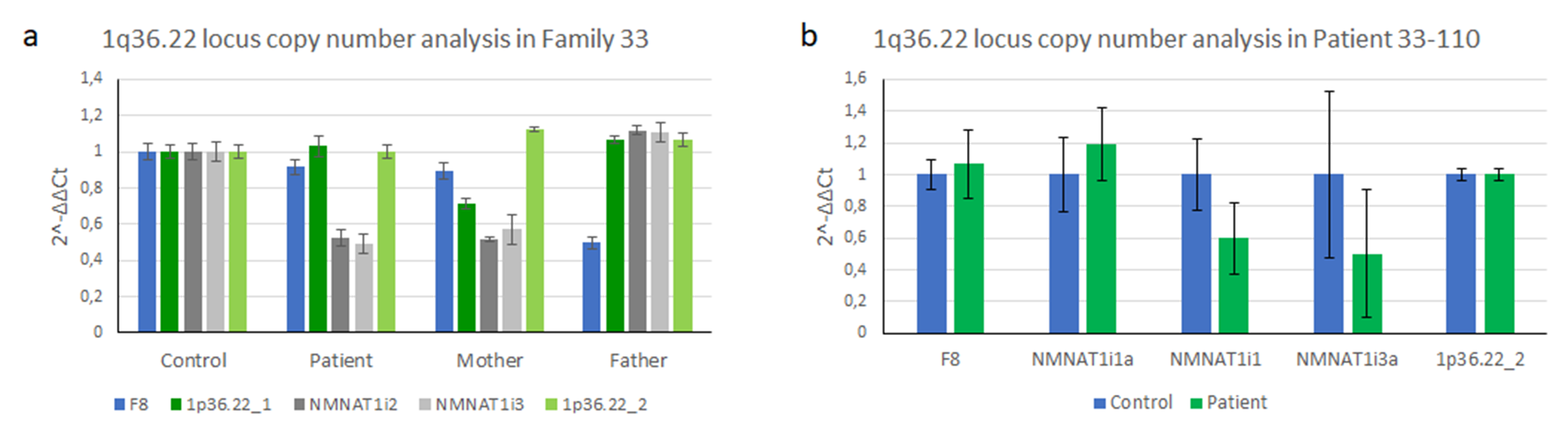

Supplement: Supplementary file 4 — High resolution image (TIF 1585 kb) [file 13353_2022_733_MOESM2_ESM.tif]
