# Supplementary material for: Molecular background of Leber congenital amaurosis in a Polish cohort of patients—novel variants discovered by NGS
Source: J Appl Genet. 2022 Nov 12;64(1):89–104. doi: 10.1007/s13353-022-00733-9 (PMC9837007; doi:10.1007/s13353-022-00733-9)
Supplement: Supplementary file 5 — (DOCX 15 kb) [file 13353_2022_733_MOESM3_ESM.docx]

**Supplementary material 1**

**Supplementary Material 1a. List of genes associated with inherited retinal dystrophies analyzed on WES**

*ABCA4, ABCC6, ABHD12, ACBD5, ACO2, ADAM9, ADAMTS18, ADGRA3, ADGRV1, ADIPOR1, AFG3L2, AGBL5, AHI1, AHR, AIPL1, ALMS1, ARHGEF18, ARL2BP, ARL3, ARL6, ARMS2, ARSG, ASRGL1, ATF6, ATXN7, BBIP1, BBS1, BBS10, BBS12, BBS2, BBS4, BBS5, BBS7, BBS9, BEST1, C12orf65, C1QTNF5, C21orf2, C2orf71, C3, C8orf37, CA4, CABP4, CACNA1F, CACNA2D4, CAPN5, CC2D2A, CCT2, CDH23, CDH3, CDHR1, CEP164, CEP19, CEP250, CEP290, CEP78, CERKL, CFAP410, CFH, CHM, CIB2, CLCC1, CLN3, CLRN1, CLUAP1, CNGA1, CNGA3, CNGB1, CNGB3, CNNM4, COL11A1, COL2A1, COL9A1, CRB1, CRX, CSPP1, CTNNA1, CWC27, CYP4V2, DFNB31, DHDDS, DHX38, DMD, DRAM2, DTHD1, DYNC2H1, DYNC2I2, EFEMP1, ELOVL1, ELOVL4, EMC1, ENSA, ERCC6, ESPN, EXOSC2, EYS, FAM161A, FBLN5, FLVCR1, FSCN2, FZD4, GDF6, GNAT1, GNAT2, GNB3, GNPTG, GPR125, GPR179, GRK1, GRM6, GUCA1A, GUCA1B, GUCY2D, HARS, HGSNAT, HK1, HMCN1, HMX1, HTRA1, IDH3B, IFT140, IFT172, IFT27, IFT81, IMPDH1, IMPG1, IMPG2, INPP5E, INVS, IQCB1, ITM2B, JAG1, KCNJ13, KCNV2, KIAA1549, KIF11, KIF3B, KIZ, KLHL7, KSS, LAMA1, LCA5, LHON, LRAT, LRIT3, LRP5, LTBP2, LZTFL1, MAK, MAPKAPK3, MERTK, MFN2, MFRP, MFSD8, MIEF1, MIR204, MKKS, MKS1, MT-ATP6, MT-TH, MT-TL1, MTTP, MT-TP, MT-TS2, MVK, MYO7A, NBAS, NDP, NEK2, NEUROD1, NMNAT1, NPHP1, NPHP3, NPHP4, NR2E3, NR2F1, NRL, NYX, OAT, OFD1, OPA1, OPA3, OPN1LW, OPN1MW, OPN1SW, OTX2, PANK2, PAX2, PCDH15, PCYT1A, PDE6A, PDE6B, PDE6C, PDE6G, PDE6H, PDZD7, PEX1, PEX2, PEX7, PGK1, PHYH, PITPNM3, PLA2G5, PLK4, PNPLA6, POC1B, POC5, POCS, POMGNT1, PRCD, PRDM13, PRKCG, PROM1, PROS1, PRPF3, PRPF31, PRPF4, PRPF6, PRPF8, PRPH2, PRPS1, RAB28, RAX2, RB1, RBP3, RBP4, RCBTB1, RD3, RDH11, RDH12, RDH5, REEP6, RGR, RGS9, RGS9BP, RHO, RIMS1, RLBP1, ROM1, RP1, RP1L1, RP2, RP9, RPE65, RPGR, RPGRIP1, RPGRIP1L, RS1, RTN4IP1, SAG, SAMD11, SDCCAG8, SEMA4A, SLC24A1, SLC25A46, SLC4A7, SLC7A14, SNRNP200, SPATA7, SPP2, TEAD1, TIMM8A, TIMP3, TLR3, TLR4, TMEM126A, TMEM216, TMEM237, TOPORS, TREX1, TRIM32, TRNT1, TRPM1, TSPAN12, TTC8, TTC8R, TTLL5, TTPA, TUB, TUBGCP4, TUBGCP6, TULP1, UNC119, USH1C, USH1G, USH2A, VCAN, VPS13B, WDPCP, WDR19, WFS1, ZNF408, ZNF423, ZNF513*

**Supplementary material 1b. List of genes analyzed on NGS retinal diagnostic panel**

*ABCA4, ABCC6, ABHD12, ACBD5, ACO2, ADAM9, ADAMTS18, ADGRA3, ADGRV1, ADIPOR1, AFG3L2, AGBL5, AHI1, AHR, AIPL1, ALMS1, ARHGEF18, ARL2BP, ARL3, ARL6, ARMS2, ARSG, ASRGL1, ATF6, ATXN7, BBIP1, BBS1, BBS10, BBS12, BBS2, BBS4, BBS5, BBS7, BBS9, BEST1, C12orf65, C1QTNF5, C2orf71, C3, C8orf37, CA4, CABP4, CACNA1F, CACNA2D4, CAPN5, CC2D2A, CCT2, CDH23, CDH3, CDHR1, CEP164, CEP19, CEP250, CEP290, CEP78, CERKL, CFAP410, CFH, CHM, CIB2, CLCC1, CLN3, CLRN1, CLUAP1, CNGA1, CNGA3, CNGB1, CNGB3, CNNM4, COL11A1, COL2A1, COL9A1, CRB1, CRX, CSPP1, CTNNA1, CYP4V2, DFNB31, DHDDS, DHX38, DMD, DRAM2, DTHD1, EFEMP1, ELOVL1, ELOVL4, EMC1, ERCC6, ESPN, EXOSC2, EYS, FAM161A, FBLN5, FLVCR1, FSCN2, FZD4, GDF6, GNAT1, GNAT2, GNB3, GNPTG, GPR179, GRK1, GRM6, GUCA1A, GUCA1B, GUCY2D, HARS, HGSNAT, HK1, HMCN1, HMX1, HTRA1, IDH3B, IFT140, IFT172, IFT27, IFT81, IMPDH1, IMPG1, IMPG2, INPP5E, INVS, IQCB1, ITM2B, JAG1, KCNJ13, KCNV2, KIAA1549, KIF11, KIZ, KLHL7, LAMA1, LCA5, LRAT, LRIT3, LRP5, LTBP2, LZTFL1, MAK, MAPKAPK3, MERTK, MFN2, MFRP, MFSD8, MKKS, MKS1, MTTP, MVK, MYO7A, NBAS, NDP, NEK2, NEUROD1, NMNAT1, NPHP1, NPHP3, NPHP4, NR2E3, NR2F1, NRL, NYX, OAT, OFD1, OPA1, OPA3, OPN1LW, OPN1MW, OPN1SW, OTX2, PANK2, PAX2, PCDH15, PCYT1A, PDE6A, PDE6B, PDE6C, PDE6G, PDE6H, PDZD7, PEX1, PEX2, PEX7, PGK1, PHYH, PITPNM3, PLA2G5, PLK4, PNPLA6, POC1B, POCS, POMGNT1, PRCD, PRDM13, PRKCG, PROM1, PRPF3, PRPF31, PRPF4, PRPF6, PRPF8, PRPH2, PRPS1, RAB28, RAX2, RB1, RBP3, RBP4, RCBTB1, RD3, RDH11, RDH12, RDH5, REEP6, RGR, RGS9, RGS9BP, RHO, RIMS1, RLBP1, ROM1, RP1, RP1L1, RP2, RP9, RPE65, RPGR, RPGRIP1, RPGRIP1L, RS1, RTN4IP1, SAG, SAMD11, SDCCAG8, SEMA4A, SLC24A1, SLC25A46, SLC7A14, SNRNP200, SPATA7, SPP2, TEAD1, TIMM8A, TIMP3, TLR3, TLR4, TMEM126A, TMEM216, TMEM237, TOPORS, TREX1, TRIM32, TRNT1, TRPM1, TSPAN12, TTC8, ttc8r, TTLL5, TTPA, TUB, TUBGCP4, TUBGCP6, TULP1, UNC119, USH1C, USH1G, USH2A, VCAN, VPS13B, WDPCP, WDR19, WFS1, ZNF408, ZNF423, ZNF513.*
