# Supplementary material for: Molecular background of Leber congenital amaurosis in a Polish cohort of patients—novel variants discovered by NGS
Source: J Appl Genet. 2022 Nov 12;64(1):89–104. doi: 10.1007/s13353-022-00733-9 (PMC9837007; doi:10.1007/s13353-022-00733-9)
Supplement: Supplementary file 6 — (DOCX 19 kb) [file 13353_2022_733_MOESM4_ESM.docx]

**Supplementary Table 2**

Primer pairs and size of PCR products used for Sanger sequencing in this study

| **Gene symbol** | **Primer name** | **Primer sequence (5’-3’)** | **Product size (pz)** |
| --- | --- | --- | --- |
| *CEP290* | CEP290e5F  CEP290e5R | 5’tggatctgccatataatactttttgt3’  5’tcacaaccatatgctcagtcct3’ | 386 |
|  | CEP290e13partF  CEP290e13partR | 5’atcattgtgtattacagttgaagtgt3’  5’ataaaaatacCTTTGTTTCTTTGGAGC3’ | 301 |
|  | CEP290e15end_extra_F  CEP290e15end_R | 5’TGCTGTCGTTGAAATAAAGAATTGT3’ 5’tccacttcaatcaggttagctcc3’ | 310 |
|  | CEP_e18F  CEP_e18R | 5’TTTGGAGGGATTTTGGAAAC3’ 5’TGTTCAAGAATGAGATAAAGGGG3’ | 640 |
|  | CEP_intr26F  CEP_intr26R | 5’ATCTTGGCTCACTGCAAGCT3’  5’ACTGTGGTCAGAAAACTCAGCT3’ | 531 |
|  | CE290e35_F  CE290e35_R | 5’tcagatgaagcattttaaagggaaa3’ 5’aaaactaatggataacagcataactca3’ | 347 |
|  | CEP290e37end_new_F  CEP290e37end_new_R | 5’AGCATTTTATTCGTCTGGCTGA3’ 5’actagtttcaaattatggctttccc3’ | 401 |
|  | CEP_e46F  CEP_e46R | 5’acgtcttagttcaaaatcttccttgt3’  5’catttctggcttatcactgctga3’ | 302 |
| *CRB1* | CRB1e6part_extra_F  CRB1e6part_extra_R | 5’gcacttctgcaagattatacaagt3’  5’TTGACCCACAGGAAGCCATC3’ | 411 |
|  | CRB1e6middleF  CRB1e6middleR | 5’GGAGCTGCTAAGTGGCTACA3’  5’AACGAAGGTGTGGATGGCAT3’ | 312 |
|  | CRB1e6end_F  CRB1e6endF | 5’TAATATGCCATCCACACCTTCGT3’ 5’ttttgctgtttctgctctgc3’ | 301 |
|  | CRB1_e9end_F  CRB1_e9end_R | 5’CTGCAAGGGTGTCTAAGTACA3’  5’CCAAGGGACAGGAGCAATGA3’ | 226 |
|  | CRB1e9part_new_F  CRB1e9part_new_R | 5’ccatcccagtttgatattctggc3’ 5’GCATCCCTTGTTCTGAAACCA3’ | 406 |
| *CRX* | CRXe4_partF  CRXe4_partR | 5’CTCAGGCTCCCCAACCAC3’  5’ACTGGGCCAGGGAAGGTC3’ | 299 |
| *GUCY2D* | GUCY2De2end_F  GUCY2De2end_R | 5’AGCTGCTCGCCGAAGAAG3’ 5’ggacagaggcttggctcg3’ | 391 |
|  | GUCY2D_e11F  GUCY2D_e11R | 5’ctcaggttgcagggtctcag3’ 5’agatgccagctttaaggggg3’ | 414 |
|  | GUCY2D_e12F  GUCY2D_e12R | 5’CTCTCCCTCCACACACACAC3’  5’TGGAAGGCCAGAGGTCCTG3’ | 414 |
|  | GUCY2D_e14F  GUCY2D_e14R | 5’GCTGCTTACACAGATGCTGC3’  5’GUCY2De14R: agctggggactggaggaata3’ | 462 |
| *LCA5* | LCA5e8_partF  LCA5e8_partR | 5’TGGATAAATTGCAAGGAGAGGA3’  5’TCCATACTGTTTCTTTGGAAATCCA3’ | 403 |
| *LRAT* | LRATe2part_F  LRATe2part_R | 5’agcttaacttgcccagccc3’ 5’CTGTCAGGGCCAACAGGATG3’ | 320 |
| *NMNAT1* | NMNAT1e3_F  NMNAT1e3_R | 5’AGGTACACAGTTGTCAAAGGCA3’  5’gccgcttagccatttacacag3’ | 223 |
|  | NMNAT1_e5_newF  NMNAT1_e5_newR | 5’GGAGCAACATTCACGTGGTG3’  5’AGGCAACAGATCACAACTTCT3’ | 315 |
| *RPGRIP1* | RPGRIP1e17part_F  RPGRIP1e17part_R | 5’tccagttgggatagctgttctc3’  5’GCAAGAGGCAGTAAAGGCAC3’ | 439 |

For all the amplified fragments the following PCR conditions were applied: 95°C, 3 min (preliminary denaturation); 40 cycles of denaturation at 94°C for 30 s, annealing for 30 s with temperature starting from 63°C, decreasing to 55°C (touchdown PCR −0.2°C per cycle), elongation at 72°C for 45 s; and the final synthesis at 72°C, 10 min.
