# Supplementary material for: Molecular background of Leber congenital amaurosis in a Polish cohort of patients—novel variants discovered by NGS
Source: J Appl Genet. 2022 Nov 12;64(1):89–104. doi: 10.1007/s13353-022-00733-9 (PMC9837007; doi:10.1007/s13353-022-00733-9)
Supplement: Supplementary file 7 — (DOCX 18 kb) [file 13353_2022_733_MOESM5_ESM.docx]

**Supplementary Table 2.** Oligonucleotide primers used to perform copy number analysis of the 1p36.22 region in Family no. 33.

| **Primer name** | **sequence (5'->3')** | **Genomic coordinates (hg38)** | **Target** | **Copy number** |
| --- | --- | --- | --- | --- |
| **Reference gene** | | | | |
| ALB_F | TGAAATGGCTGACTGCTGTG | chr4:73408650-73408732 | Albumin (*ALB*) | Normal |
| ALB_R | GGAGGTTTGGGTTGTCATCT |  |  |  |
| **Sex determination** | | | | |
| F8_F | TTTCCATTCAACACCTCAGTCGT | chrX:154999491-154999575 | Factor VIII (*F8*) | Normal |
| F8_R | GCCTTGGCTTAGCGATGTTG |  |  |  |
| **locus of interest** | | | | |
| 1p36.22_F1 | TGCCACACACTAAGCAGGAG | chr1:9415856-9415942 | 1p36.22 | Normal |
| 1p36.22_R1 | CCAGAAACATTTGGCAGGAT |  |  |  |
| NMNAT1e1_F | CACAGAAATCACACCTCCAC | chr1:9943472-9943556 |  |  |
| NMNAT1e1_R | ACGTTCTGTGAGGAAAACAG |  |  |  |
| NMNAT1i1b_F | TAGGGAAAATGCTGGGACAG | chr1:9946502-9946586 |  |  |
| NMNAT1i1b_R | AAGAGGCCCAAGTGTTCAGA |  |  |  |
| NMNAT1i1c_F | AACTGAAGGATCCCACACCA | chr1:9952383-9952462 |  |  |
| NMNAT1i1c_R | TGTGCAGTTTGTTCAGATGGA |  |  |  |
| NMNAT1i1d_F | GCCTCAAAGTGTTGGGGTTA | chr1:9954464-9954546 |  |  |
| NMNAT1i1d_R | CAAGGACAATGGCAACTTGA |  |  |  |
| NMNAT1i1e_F | GCGTAACACCAGAAGGCACT | chr1:9955965-9956044 |  |  |
| NMNAT1i1e_R | GTGAGTTGCCTACAGCCAAGA |  |  |  |
| NMNAT1i1a_F | CCTCAGCCCTGCAGACATAA | chr1:9957908-9957997 |  |  |
| NMNAT1i1a_R | AGACAATGAGGGGACCAATG |  |  |  |
| NMNAT1i1_F | TGTTGGCTTGACGATCTGAG | chr1:9970990-9971077 |  | Deletion |
| NMNAT1i1_R | CGACCAGCCAACTACAGTCC |  |  |  |
| NMNAT1i2_F | CACAAACATGCTGTCCTGCT | chr1:9972566-9972651 |  |  |
| NMNAT1i2_R | GCCAGATGCTCAATTCTTCC |  |  |  |
| NMNAT1i3_F | GCCATTCCTGTGGAAGGTTA | chr1:9975903-9975984 |  |  |
| NMNAT1i3_R | TGAAAGACACAGAGGGGCTAA |  |  |  |
| NMNAT1i3b_F | AGTGGACTCCAGCGTCTAGC | chr1:9977999-9978081 |  |  |
| NMNAT1i3b_R | CCCGGCCTGATCCTATTATT |  |  |  |
| NMNAT1i3c_F | ATGGCCTCTGTCCTCCTTCT | chr1:9978397-9978480 |  |  |
| NMNAT1i3c_R | AGCAGGCTGTGGACTTGACT |  |  |  |
| NMNAT1i3a_F | CCGCTCTAGGACCTTCTCCT | chr1:9978891-9978973 |  |  |
| NMNAT1i3a_R | GAGCCCACACTCTTGACCAT |  |  |  |
| 1p36.22_F2 | CCTTTTGACCTCCCTGTTCA | chr1:10415188-10415268 |  | Normal |
| 1p36.22_R2 | AACCACCGACTTTCCTCCTT |  |  |  |
